# Large Language Models Versus Clinicians in Predicting Recovery from Idiopathic Sudden Sensorineural Hearing Loss

**DOI:** 10.3390/diagnostics16213646

**Published:** 2026-11-04

**Authors:** Rezarta Taga-Senirli, Ahmet Harper, Mesut Güneş, Yeşim Yüksel, Muhammet Yıldız, Nuray Ensari, Özer Erdem Gür

**Affiliations:** Department of Otolaryngology, Antalya Training and Research Hospital, Antalya 07070, Türkiye; harperrrahmet@gmail.com (A.H.); mesut.gunes.1976@gmail.com (M.G.); yesimgedikli@gmail.com (Y.Y.); dr_yildiz_muhammet@hotmail.com (M.Y.); nurayakmese@yahoo.com (N.E.); erdemkaptan@yahoo.com (Ö.E.G.)

**Keywords:** sudden sensorineural hearing loss, artificial intelligence, large language models, prognosis, ChatGPT, hearing recovery

## Abstract

**Background/Objectives**: Early prognostication in idiopathic sudden sensorineural hearing loss (SSNHL) remains difficult, and although the use of large language models (LLMs) in otolaryngology is being increasingly evaluated, data directly comparing their prognostic predictions with those of clinicians assessing the same patients remain limited. This study evaluated the zero-shot, role-prompted performance of three contemporary LLMs against a panel of board-certified otolaryngologists in forecasting SSNHL outcomes. **Methods:** We retrospectively evaluated 100 consecutive patients with idiopathic SSNHL and randomly selected 30 for a blinded observer comparison. In the primary cohort of 100 patients, recovery was classified as favorable recovery (complete/partial, *n* = 37, 37.0%) or poor outcome (slight/none, *n* = 63, 63.0%) based on functional audiometric utility. Identical de-identified multimodal case sets (*n* = 30; 9 favorable, 21 poor), including the baseline audiogram image, were independently graded for 1-month recovery likelihood (low/moderate/high) by three otolaryngologists and three zero-shot LLMs—ChatGPT (GPT-5.6 Sol; OpenAI), Gemini (Gemini 3.1 Pro; Google), and Claude (Claude Opus 4.7; Anthropic), accessed through their publicly available web interfaces in July 2026—all blinded to outcomes; actual recovery was classified using the Siegel criteria. **Results:** In the primary cohort, multivariable logistic regression identified baseline pure-tone average (aOR = 0.957, 95% CI: 0.936–0.979, *p* = 0.0001) and admission delay (aOR = 0.797, 95% CI: 0.656–0.966, *p* = 0.0211) as significant independent predictors of recovery (model C-statistic = 0.834, Hosmer–Lemeshow *p* = 0.7925). In the sub-cohort (*n* = 30), Expert 2 achieved the highest overall diagnostic accuracy (86.7%, 95% CI: 70.3–94.7%; sensitivity 95.2%, specificity 66.7%). All three LLMs achieved identical overall accuracy (70.0%) that did not differ significantly from that of individual experts, although the study was not powered to establish equivalence, and all models remained below the best-performing expert (86.7%). Gemini achieved the highest specificity (77.8%, 95% CI: 45.3–93.7%) and the highest positive predictive value (87.5%, 95% CI: 64.0–96.5%) among all raters, and every case it rated as having low recovery potential failed to recover (10 of 10). **Conclusions:** Zero-shot, role-prompted LLMs predicted 1-month SSNHL outcome with 70.0% accuracy—not significantly different from that of individual experienced otolaryngologists, though the study was not powered for equivalence—and below the best-performing expert. Their reliable “rule-in” but poor “rule-out” pattern warrants prospective evaluation and does not yet support use in treatment decisions.

## 1. Introduction

Sudden sensorineural hearing loss (SSNHL) is an otologic emergency, conventionally defined as sensorineural hearing loss of at least 30 dB across three contiguous audiometric frequencies that develops within 72 h [1]. Although a specific etiology—such as vestibular schwannoma, autoimmune disease, or vascular compromise—is identified in a minority of cases, approximately 90% remain idiopathic despite appropriate work-up [1]. The reported incidence has traditionally ranged from 5 to 20 cases per 100,000 persons per year, but contemporary population-based estimates with more complete case ascertainment place it considerably higher—27 per 100,000 in a large United States claims analysis [2], and up to 160 per 100,000 in a German population-based study [3]—with a peak in the fifth and sixth decades of life. Because timely treatment—most commonly systemic and/or intratympanic corticosteroids, with hyperbaric oxygen therapy as an adjunct in selected cases—is thought to be most effective within the first two weeks, SSNHL is managed as a time-sensitive condition [1].

Despite this urgency, the natural history of SSNHL is strikingly heterogeneous: spontaneous recovery occurs in roughly 32% to 65% of untreated patients, while a substantial subset is left with permanent, disabling hearing loss [1]. Several prognostic factors have been repeatedly associated with poorer recovery—older age, the presence of vertigo, a greater degree of initial hearing loss, a downsloping or profound/total audiometric configuration, delayed presentation, and, more recently, systemic inflammatory markers such as the neutrophil-to-lymphocyte ratio [4,5]. However, these factors only partially explain outcome and interact in complex ways, so accurate prognosis at the level of the individual ear remains difficult and, in practice, is often based on the subjective, experience-driven judgment of the treating clinician [1,6]. This uncertainty carries direct therapeutic consequences: an ear reliably recognized at presentation as having a poor prognosis might benefit from earlier and more aggressive intervention—for example, up-front combination or intratympanic salvage therapy—before the therapeutic window closes.

Over the past decade, artificial intelligence (AI), and machine learning (ML) in particular, has found broad application across otolaryngology–head and neck surgery, including audiometric interpretation, otologic and imaging diagnostics, head and neck oncology, and outcome prediction [6,7,8,9]. Within this trend, several groups have specifically developed ML and deep-learning models to predict hearing recovery in SSNHL using routinely available demographic, audiometric, and laboratory data, with model performance often driven by the initial audiogram shape and the degree of hearing loss—the variables that clinicians weight most heavily [10,11,12]. Some of these models exhibit good discriminative performance, suggesting that AI could serve as a decision-support tool at the point of care.

An important practical question, however, concerns how such models perform relative to experienced clinicians confronted with the same patient at presentation. Recent studies have begun to examine LLMs in the context of SSNHL—including treatment decision-making [13], patient-oriented education [14], and multi-model question-answering performance [15]—yet direct, case-level benchmarking of LLM prognostication against expert human judgment remains scarce, particularly for the clinically decisive task of identifying, early and reliably, the ear that is unlikely to recover.

Accordingly, the present study compares the prognostic performance of contemporary AI models with that of clinicians in predicting recovery from SSNHL. Our central aim is not to replace clinical judgment but to test whether AI models can reliably flag the poor-prognosis ear at presentation; such a capability, if confirmed in prospective studies, would warrant evaluation as a trigger for earlier treatment intensification in patients most likely to benefit from it.

## 2. Materials and Methods

### 2.1. Patient Selection, Randomization, and Ethical Framework

This retrospective study was conducted in accordance with the Declaration of Helsinki and approved by the Institutional Ethics Committee of Antalya Training and Research Hospital (approval No. 12/7, dated 08 July 2026); owing to the retrospective design and the use of anonymized data, the requirement for study-specific informed consent was waived.

We reviewed patients who presented with sudden hearing loss at the Department of Otorhinolaryngology of Antalya Training and Research Hospital, a tertiary referral center, between January 2025 and January 2026 and were diagnosed with idiopathic sudden sensorineural hearing loss (SSNHL). The inclusion criteria were as follows: (1) subjective hearing loss with sudden onset (<72 h); (2) sensorineural hearing loss of ≥30 dB across at least three contiguous frequencies documented by baseline pure-tone audiometry (PTA); (3) age ≥10 years; and (4) complete clinical, demographic, laboratory, and 1-month follow-up audiometric data. Patients with active middle-ear disease, vestibular schwannoma (excluded by magnetic resonance imaging), bilateral sudden hearing loss, or a history of fluctuating hearing loss (e.g., Ménière disease) were excluded.

During the study period, 100 consecutive patients met these criteria and constituted the primary cohort. To establish a rigorous comparative benchmark while managing cognitive burden on clinical experts, a sub-cohort of 30 patients was extracted using computer-generated simple random sampling from this cohort (SAS RAND function). The validation sub-cohort comprised 21 poor-outcome cases (70.0%) and 9 favorable-recovery cases (30.0%), closely approximating the primary cohort.

The restriction of the observer-comparison analysis to 30 patients was justified on both practical and statistical grounds. Blinded, in-depth grading of complete case sets—each comprising the full structured clinical and laboratory profile together with the baseline audiogram—by three independent clinicians across all 100 patients would have imposed an unsustainable evaluator burden and risked degrading the quality and consistency of the ratings; the same constraint applied to the individually audited, single-session evaluations of the three AI models. Statistically, a sample of 30 patients provides 80% power at a two-tailed α of 0.05 to detect prediction–outcome correlation coefficients of r ≥ 0.45. Finally, expert panels of 20–30 cases are an established methodological precedent in medical artificial-intelligence validation studies assessing inter-rater reliability, balancing statistical robustness against the clinical depth of individual case evaluations.

### 2.2. Clinical Parameters and Treatment Protocols

A comprehensive profile was extracted for each patient and recorded in a structured spreadsheet, comprising the following: demographic and lifestyle data (age, sex, body mass index, smoking, and alcohol use); time from symptom onset to admission (days); affected side; associated audio-vestibular symptoms (tinnitus, vertigo, dizziness, otalgia); relevant otologic and medical history (previous SSNHL episode, prior ear surgery, head/temporal trauma, acoustic trauma, ototoxic drug use); systemic comorbidities (hypertension, diabetes mellitus, hyperlipidemia, cardiovascular disease, cerebrovascular accident, chronic kidney disease, thyroid disease, autoimmune disease, and anxiety/depression); and laboratory parameters (complete blood count with hemoglobin, hematocrit, white-blood-cell, neutrophil, lymphocyte, monocyte, and platelet counts and the derived neutrophil-to-lymphocyte ratio [NLR] and platelet-to-lymphocyte ratio [PLR]; prothrombin time, activated partial thromboplastin time, and international normalized ratio; uric acid, creatinine, aspartate and alanine aminotransferases, and glucose; triglycerides and low- and high-density lipoprotein cholesterol; and thyroid-stimulating hormone, T3, and T4).

Standardized treatment consisted of primary systemic corticosteroid therapy (oral methylprednisolone, 1 mg/kg/day, tapered over 10–14 days), administered with or without concurrent hyperbaric oxygen therapy (HBOT; 2.4 atmospheres absolute for 120 min per session over 10–20 sessions) and/or intratympanic dexamethasone (4 mg/mL, 3–5 sessions, as concurrent or salvage therapy). The specific combination administered to each patient was recorded.

### 2.3. Input Dataset and Observer Evaluation

For every patient, an identical, de-identified case set was prepared, comprising the complete structured clinical and laboratory profile described above together with a digital image of the patient’s baseline (presentation) audiogram. The cases were presented to all evaluators (both human and AI) in the same randomized sequence, labeled using sequential identifiers (patient 1, patient 2, …, patient 30) to preserve anonymity.

The case sets were assessed independently and blindly by three board-certified otolaryngologists. None of the evaluators had been involved in the patients’ treatment, and all were blinded to patient identity and to the actual clinical outcomes.

All six evaluators (three human, three AI) categorized the likelihood of hearing recovery at 1 month into one of three ordinal grades: 1 (low), 2 (moderate), or 3 (high).

### 2.4. Multi-Rater Human Expert Panel and LLM Evaluation Protocol

Actual hearing recovery was determined at 1 month post-treatment—the interval of greatest therapeutic relevance—using Siegel’s classic criteria: complete recovery (final PTA <25 dB), partial recovery (final PTA 25–45 dB with >15 dB gain), slight recovery (final PTA >45 dB with >15 dB gain), and no recovery (<15 dB gain) [16]. For binary analysis, ears with slight or no recovery were classified as non-recovered, and those with partial or complete recovery as recovered, consistent with the outcome definitions of previous machine-learning studies of SSNHL prognosis [11,12]. Hearing gain was additionally analyzed as a continuous variable, calculated as the difference between baseline and 1-month PTA thresholds (PTA_baseline − PTA_1 month), with positive values indicating objective improvement.

The 30 cases were independently evaluated by three board-certified otolaryngologists, all holding academic appointments as associate professors: two with more than 10 years of post-residency experience (Experts 1 and 2) and one with more than 15 years of post-residency experience and subspecialty expertise in otology (Expert 3).

Three commercial large language models were evaluated in July 2026: ChatGPT (GPT-5.6 Sol; OpenAI), Claude (Claude Opus 4.7; Anthropic), and Gemini (Gemini 3.1 Pro; Google). All models were accessed through their publicly available web interfaces using default settings; generation parameters such as temperature are not user-configurable in this setting and were therefore not modified.

All evaluators—human and model—received the same anonymized case information: age, sex, body mass index, comorbidities, systemic inflammatory markers (neutrophil-to-lymphocyte and platelet-to-lymphocyte ratios), time from symptom onset to admission, the baseline pure-tone audiogram (provided as an image), and the treatment administered, reported at the protocol level (without dose or duration details). The prompt (originally in Turkish but translated here) was as follows: “Act as an otolaryngologist with at least 10 years of experience who follows the current literature. I will provide the clinical and laboratory information of patients presenting with sudden hearing loss, obtained from history-taking after presentation and ask you to predict the likelihood of recovery of their hearing loss.” The full variable set and the baseline audiogram image were then supplied for each case. The prediction task was therefore framed as prognosis at treatment initiation: estimating the 1-month hearing outcome given the complete clinical presentation and the therapy received. No post-treatment information of any kind (follow-up audiometry, clinical response) was provided, and the human experts were additionally blinded to one another’s ratings.

For each model, the evaluation was performed in a single continuous session initiated as a fresh conversation, so that no prior, unrelated conversational context could influence the responses; the 30 cases were then presented sequentially within this session using an identical standardized prompt for every case. The prompt instructed each model to respond as an otolaryngologist with at least 10 years of clinical experience; this role instruction was chosen deliberately to match the experience level of the human expert panel, thereby standardizing the clinical perspective across human and model evaluators. The complete prompt (original Turkish with English translation) is provided in the Supplementary Materials. Because the behavior of commercial LLMs may change across model versions, the exact model versions and the evaluation date are reported to support reproducibility.

### 2.5. Statistical Analysis and Model Diagnostics

Inter-observer agreement among the six evaluators was assessed on a prognostic scale of 1–3 using linear and quadratic weighted kappa (κw). The concordance between each evaluator’s predictions and the actual 1-month outcomes was evaluated by Spearman rank correlation between the 3-level prediction and both the 4-level Siegel outcome and the continuous hearing gain.

The primary clinical endpoint—early identification of the ear unlikely to recover—was analyzed in a binary framework. For each evaluator, the distribution of the 3-level prognostic ratings across the observed binary outcomes (recovered vs. non-recovered) was first compared using Fisher’s exact test. Each evaluator’s prediction was then dichotomized as poor (grade 1–2, low or moderate recovery chance) versus favorable (grade 3, high recovery chance) and tested against actual non-recovery, with sensitivity, specificity, positive and negative predictive values, and overall accuracy calculated for each evaluator.

Statistical analyses were executed using SAS software (Version 9.4, SAS Institute Inc., Cary, NC, USA) and Python 3.12. Continuous variables were compared using Mann–Whitney U tests, and categorical proportions using Fisher’s exact test. Multivariable logistic regression model performance was evaluated using the C-statistic (AUC), a Hosmer-Lemeshow goodness-of-fit test, Variance Inflation Factors (VIFs) for multicollinearity, and Events Per Variable (EPV) ratios. Diagnostic performance metrics (sensitivity, specificity, accuracy, PPV, NPV) were computed with 95% Confidence Intervals using the Wilson Score. Inter-evaluator agreement was tested using paired McNemar tests. Statistical significance was set at *p* < 0.05. Detailed graphical diagnostic evaluations, including the calibration curve, ROC curve, Cook’s distance influence plot, and leverage residuals, are illustrated in Supplementary Figure S1.

## 3. Results

### 3.1. Baseline Patient Demographics and Clinical Characteristics

The characteristics of the full cohort (*N* = 100) and the observer sub-cohort (*n* = 30) are summarized in Table 1. All 100 enrolled patients had complete clinical profiles and follow-up data. The full cohort had a mean age of 49.6 ± 13.8 years and consisted of 49 (49.0%) women and 51 (51.0%) men; the mean interval from symptom onset to presentation was 4.8 ± 4.6 days (range, 1–22). Hypertension was present in 21 patients (21.0%) and diabetes mellitus in 19 (19.0%). All patients received oral systemic corticosteroid therapy; 90 (90.0%) additionally received hyperbaric oxygen therapy (HBOT) and 21 (21.0%) received adjunctive intratympanic steroid injections. At 1 month, the Siegel criteria classified 27 patients as completely recovered, 10 as partially recovered, 11 as slightly recovered, and 52 as not recovered; under the binary classification, 37 patients (37.0%) were classified as recovered (complete or partial) and 63 (63.0%) as non-recovered.

The observer sub-cohort comprised 21 (70.0%) women and 9 (30.0%) men, with a mean age of 48.2 ± 13.2 years (range, 24–70) and a mean admission delay of 3.9 ± 3.3 days (range, 1–16). Six patients (20.0%) had hypertension and five (16.7%) diabetes mellitus. All patients received systemic corticosteroids, with 21 (70.0%) additionally receiving HBOT and 5 (16.7%) intratympanic steroids. The Siegel criteria classified 6 patients (20.0%) as completely recovered, 3 (10.0%) as partially recovered, 2 (6.7%) as slight, and 19 (63.3%) as no recovery; in the binary classification, 9 patients (30.0%) were classified as recovered and 21 (70.0%) as non-recovered. Among the 27 patients with valid paired audiometric data, the mean hearing gain was 10.3 ± 16.6 dB (range, −17.0 to 68.0). Baseline characteristics did not differ significantly between the sub-cohort and the full cohort (Table 1), consistent with representative random sampling.

### 3.2. Inter-Observer Agreement

The complete matrix of pairwise linearly weighted kappa (κw) coefficients among the six evaluators is displayed in Table 2. The highest pairwise agreement in the matrix was observed between ChatGPT and Expert 1 (κw = 0.720; 95% CI, 0.540–0.900; substantial agreement), followed by Gemini and Expert 1 (κw = 0.641). The three AI models showed consistent agreement with one another (ChatGPT–Gemini κw = 0.667; ChatGPT–Claude κw = 0.658; Gemini–Claude κw = 0.665). Agreement within the clinician group was lower (κw = 0.293–0.541). Expert 2 showed the lowest agreement with all other evaluators (κw range, 0.263–0.505), with the weakest pair in the matrix being Expert 2–Claude (κw = 0.263).

### 3.3. Concordance Between Predictions and Actual Outcomes

Spearman rank correlations between each evaluator’s three-level prognostic grade and the actual 1-month outcomes are summarized in Table 3 and Figure 1. Against the 4-level Siegel outcome (Figure 1A), Expert 2 showed the strongest correlation (r = 0.655, *p* < 0.001). Among the AI models, Gemini showed the highest correlation (r = 0.463, *p* = 0.010), comparable to Expert 1 (r = 0.485, *p* = 0.007); ChatGPT also reached statistical significance (r = 0.373, *p* = 0.042). The correlations of Claude (r = 0.284, *p* = 0.128) and Expert 3 (r = 0.191, *p* = 0.313) were not statistically significant. Against the continuous hearing gain (Figure 1B), only Expert 2 achieved a statistically significant correlation (r = 0.461, *p* = 0.015); no other evaluator reached significance (highest among the AI models: Gemini, r = 0.308, *p* = 0.118).

### 3.4. Prediction of Non-Recovery: Clinicians Versus AI Models

In the observer sub-cohort, 21 patients (70.0%) had an actual poor outcome (slight or no recovery) and 9 (30.0%) a favorable outcome (complete or partial recovery). Table 4 presents the cross-tabulation of prognostic predictions across the three-tier rating scale (low, moderate or high recovery potential) against observed functional outcomes. Both Expert 2 (*p* = 0.0006) and Expert 1 (*p* = 0.0152) demonstrated highly significant associations with the observed outcomes. Among the AI models, Gemini was the only one to achieve statistical significance (*p* = 0.0304); notably, all 10 cases it rated as having low recovery potential failed to recover.The diagnostic performance of all raters is summarized in Figure 2 (see also Table 5).

Table 5 outlines the diagnostic metrics for predicting poor outcome (low/moderate prediction) with Wilson Score 95% Confidence Intervals and paired McNemar comparisons. Expert 2 achieved the highest overall accuracy (86.7%, 95% CI: 70.3–94.7%), significantly outperforming Expert 3 (60.0%, McNemar *p* = 0.0269). All three LLMs demonstrated identical overall accuracies of 70.0% (95% CI: 52.1–83.3%), showing no statistically significant difference in accuracy from Expert 1 (76.7%, *p* = 0.6171–0.6831) or Expert 2 (*p* = 0.1824–0.2278). Gemini achieved the highest specificity (77.8%, 95% CI: 45.3–93.7%) and PPV (87.5%, 95% CI: 64.0–96.5%) among all raters. Given the small sub-cohort and the resulting wide confidence intervals, this absence of a statistically significant difference should not be interpreted as evidence of equivalent performance; the study was not powered for formal equivalence testing.

### 3.5. Prognostic Factors in the Full Cohort: Univariate Analysis

The baseline characteristics of the favorable- (*n* = 37) and poor-outcome (*n* = 63) groups are summarized in Table 6. Patients with a poor outcome were significantly older (52.0 ± 13.6 vs. 45.6 ± 13.3 years, *p* = 0.012) and had a higher body mass index (25.4 ± 3.1 vs. 23.0 ± 3.1 kg/m^2^, *p* < 0.001), a longer admission delay (5.8 ± 5.2 vs. 3.1 ± 2.8 days, *p* = 0.002), and a worse baseline hearing threshold (71.3 ± 26.9 vs. 46.6 ± 23.2 dB, *p* < 0.001) than those with a favorable outcome. Mean hearing gain was 26.9 ± 22.8 dB in the favorable-outcome group and 4.5 ± 20.7 dB in the poor-outcome group. Comorbidities (hypertension, diabetes mellitus, hyperlipidemia) and systemic inflammatory markers (neutrophil-to-lymphocyte and platelet-to-lymphocyte ratios) showed no significant univariate association with outcome.

### 3.6. Multivariate Analysis

In multivariate logistic regression including age, baseline hearing threshold, admission delay, NLR, and BMI (Table 7; Figure 3), baseline hearing threshold (adjusted OR, 0.957 per dB; 95% CI, 0.936–0.979; *p* < 0.001) and admission delay (adjusted OR, 0.797 per day; 95% CI, 0.656–0.966; *p* = 0.021) remained independent predictors of recovery, corresponding to 4.3% and 20.3% reductions in the odds of recovery per unit increase, respectively. BMI showed a borderline independent association (adjusted OR, 0.815; 95% CI, 0.660–1.007; *p* = 0.059), whereas age and NLR did not retain significance. One patient was excluded owing to missing laboratory data (*n* = 99). Model diagnostic testing demonstrated good performance, with C-statistic (AUC) = 0.834, Hosmer Lemeshow goodness-of-fit chi-square = 3.129 (*p* = 0.7925), and VIF < 1.65 across all terms.

### 3.7. BMI as a Predictor of Non-Recovery

In an exploratory analysis, ROC analysis of BMI for predicting non-recovery yielded an AUC of 0.717 (*p* < 0.001; Figure 4). The cutoff that maximized the Youden index in this dataset was 22.3 kg/m^2^ (sensitivity, 84.1%; specificity, 51.4%). Patients at or above this threshold had significantly higher odds of a poor outcome than those below it (OR, 5.59; 95% CI, 2.20–14.24; *p* < 0.001).

In univariate logistic modeling of recovery probability as a continuous function of BMI (intercept, 5.848; coefficient, −0.265; *p* < 0.001), the predicted probability of recovery fell below 50% at a BMI of 22.1 kg/m^2^, and below 20% above a BMI of approximately 28 kg/m^2^ (Figure 5). Because this cutoff was derived and evaluated in the same cohort, these estimates are likely optimistic and should be regarded as hypothesis-generating rather than as a clinically validated threshold.

## 4. Discussion

In this study, we directly benchmarked three generative large language models (LLMs) against three otolaryngologists in predicting 1-month hearing recovery after idiopathic sudden sensorineural hearing loss (SSNHL), using identical, de-identified case sets in a zero-shot, role-prompted setting, and we characterized the prognostic structure of the underlying 100-patient cohort. Three principal findings emerged. First, within the limits of a three-clinician benchmark, the LLMs achieved an overall accuracy (70.0%) that was descriptively intermediate between that of the human evaluators—exceeding the accuracy of one human expert (60.0%) and approaching that of a second (76.7%)—but did not reach that of the best human evaluator (86.7%). Second, agreement and accuracy dissociated: the three models agreed most strongly with each other and with Expert 1, whereas the most accurate evaluator, Expert 2, was the most divergent rater in the entire agreement matrix. Third, baseline hearing threshold and treatment delay were confirmed as independent predictors of recovery, while body mass index (BMI) emerged as a borderline independent and moderately discriminative metabolic factor—an exploratory finding that warrants confirmation in larger, prospectively designed cohorts with predefined BMI strata. Formal paired McNemar comparisons showed no statistically significant accuracy difference between the LLMs and either Expert 1 (*p* = 0.62–0.68) or Expert 2 (*p* = 0.18–0.23), whereas Expert 2 significantly outperformed Expert 3 (*p* = 0.027), the otology-subspecialized member of the panel. Given the small sub-cohort and the resulting wide confidence intervals, this absence of a statistically significant difference should not be interpreted as evidence of equivalent performance; the study was not powered for formal equivalence testing.

The dissociation between inter-observer agreement and predictive accuracy deserves emphasis. Despite originating from different developers and architectures, the three LLMs produced strikingly concordant prognostic grades (κw 0.658–0.667), and their closest human counterpart was Expert 1 (κw up to 0.720)—yet this consensus cluster was outperformed by Expert 2, whose grading agreed least with everyone else (κw 0.263–0.505). The models appear to have converged on the same “textbook” prognostic reasoning, anchored in initial audiometric severity, that dominates clinicians’ judgment and supervised machine-learning models [6,10], whereas the most accurate expert applied experience-derived thresholds that departed from this consensus. This carries a methodological warning for clinical artificial intelligence (AI) evaluation at large: agreement with expert consensus is often reported as evidence of model validity, yet a model can agree with the majority and still be systematically less accurate than the best individual clinician. Objective outcomes, not expert consensus, should remain the reference standard.

Our findings should also be viewed alongside the emerging literature on LLMs in audiology and otolaryngology, and in SSNHL specifically. Caragli et al. compared ChatGPT’s (GPT-4o) treatment recommendations with physicians’ decisions in 86 ISSNHL patients and found poor-to-fair agreement (κ −0.044 to 0.393), attributing the discordance to rigid, protocol-driven reasoning with limited contextual sensitivity [13]. Ajit-Roger et al. examined ChatGPT (ChatGPT-3.5) as a patient-education tool for SSNHL queries and reported accurate, guideline-conforming responses in both English and Spanish, with readability as the main shortcoming [14]. Aliyeva et al. submitted 27 SSNHL-related questions, including audiometric interpretation and case scenarios, to three models and found the highest expert-rated clinical accuracy for ChatGPT, with strong inter-rater reliability among ten otolaryngologist evaluators [15]. These studies evaluated treatment selection, patient communication, and question-answering quality; none assessed whether an LLM can predict the audiometrically verified outcome of an individual patient, and the model input in these studies was exclusively text-based, whereas our models additionally received the baseline audiogram as an image. Our results complement this literature from the outcome end: zero-shot, role-prompted models reached 70.0% accuracy against an objective 1-month endpoint, and—echoing the “textbook-like,” protocol-driven reasoning described by Caragli et al. [13]—their predictions clustered tightly together while diverging from the most accurate human expert. Taken together, the available evidence suggests that current LLMs reproduce consensus-level clinical reasoning across tasks: they perform well where guideline knowledge suffices (patient education, structured question-answering) and less well where individualized judgment is decisive (treatment tailoring [13] and, according to our data, matching the best individual clinician in outcome prediction).

Our results extend the literature on outcome prediction in SSNHL. Supervised machine-learning models trained on structured clinical and audiometric data have reported accuracies broadly in the 60–80% range for binary recovery prediction, with initial hearing level and audiogram configuration consistently the dominant features [10,11,12]. The present study differs fundamentally from the existing literature: the LLMs received no task-specific training, no example cases, and no cohort-specific calibration—each prediction was generated de novo from the same case file a clinician would see. The fact that zero-shot performance nonetheless matched mid-level expert accuracy suggests that general-purpose LLMs have internalized a clinically meaningful prognostic prior from their training corpora, in line with reports of LLM performance approaching clinician level in other diagnostic and prognostic tasks [17,18,19]. The ceiling observed here—whereby no model’s accuracy approached that of Expert 2—indicates that this prior reproduces average, guideline-level reasoning rather than the calibrated judgment of an outstanding individual clinician, although this interpretation rests on a small expert panel and should be regarded as descriptive rather than definitive.

The error pattern is as informative as overall accuracy. The models’ positive predictive value for non-recovery was high—with Gemini’s predictive value of 87.5% exceeding that of every human evaluator—whereas their negative predictive value was uniformly 50.0% against a 70% base rate of poor outcome. In particular, every case that Gemini rated as having low recovery potential indeed failed to recover (10 of 10), suggesting that a confident “low” flag from this model may be clinically informative, although the small number of such cases precludes drawing firm conclusions. Clinically, when a model flagged an ear as unlikely to recover, the flag was usually correct; a favorable prediction, by contrast, carried essentially no reassurance. Framed by our original aim—identifying, early and reliably, the ear that will not recover—current zero-shot LLMs are therefore better suited to “rule-in” support than to any form of de-escalation or reassurance, suggesting that a concordant model–clinician poor-prognosis flag deserves prospective evaluation as a trigger for early treatment intensification; the present data do not establish that acting on such flags improves outcomes. With identical overall accuracies of 70.0% and 50% negative predictive value, these tools cannot substitute for clinical judgment and their output should not inform treatment-intensity decisions pending prospective validation.

In the full cohort, greater baseline hearing loss and longer delay to treatment independently reduced the odds of recovery, with each day of delay lowering the odds by approximately 20%—reinforcing the management of SSNHL as an otologic emergency [1]. The more novel observation concerns BMI, which was strongly associated with non-recovery in univariate analysis, remained borderline significant after adjustment (adjusted OR 0.815 per kg/m^2^, *p* = 0.059), and discriminated non-recovery with an AUC of 0.717. A metabolic contribution to poor cochlear outcome is biologically plausible. The stria vascularis and organ of Corti depend on a terminal microcirculation with no collateral supply, rendering the cochlea particularly vulnerable to microvascular compromise; adiposity promotes endothelial dysfunction, chronic low-grade inflammation with altered adipokine signaling, dyslipidemia, and increased blood viscosity, all of which may impair cochlear perfusion and the capacity for hair-cell and synaptic recovery after an ischemic or inflammatory insult [20,21,22]. Consistent with this, higher BMI and metabolic syndrome components have been associated with both the incidence and the prognosis of SSNHL in several cohorts [23,24]. Nevertheless, we interpret the specific cutoff identified here (22.3 kg/m^2^) with deliberate caution: it lies within the conventionally normal BMI range, its specificity was modest (51.4%), and Youden-derived thresholds are cohort-dependent. We regard it as a hypothesis-generating signal that metabolic risk may operate at lower adiposity levels than commonly assumed—possibly reflecting population-specific body-composition differences—rather than as a clinically actionable threshold; external validation is required. We did not observe an association between the neutrophil-to-lymphocyte ratio and outcome; given the sample size and single-time-point measurement, this should be interpreted as an absence of evidence rather than evidence of no association, particularly as associations have been reported in larger cohorts [4,5].

Several limitations must be acknowledged. This was a retrospective, single-center study, and the observer comparison was restricted to a randomly sampled sub-cohort of 30 patients; the human benchmark comprised three otolaryngologists from a single institution, and although representative of the full cohort, the Confidence Intervals around individual diagnostic metrics are wide, and small absolute differences between evaluators should not be over-interpreted. These analyses were exploratory, and *p* values were not adjusted for multiple comparisons. Each model was evaluated in a single run with one standardized prompt. Because LLM outputs are stochastic and sensitive to prompt wording, our estimates represent one realization of each model’s behaviour; the reported confidence intervals reflect patient-level sampling uncertainty but cannot capture run-to-run or prompt-related variability. Repeated evaluations with multiple prompt formulations—feasible with version-pinned API access—are needed to quantify this additional source of uncertainty. Moreover, LLMs are continuously updated and not perfectly deterministic: the model versions tested here may not reproduce identical predictions at a later date, a reproducibility challenge that distinguishes generative AI from locked, validated prediction models. In addition, the role instruction in our standardized prompt, although chosen to mirror the experience level of the human panel, represents one specific prompting configuration; persona instructions are known to influence LLM outputs, and performance under alternative prompting strategies may differ. Recovery was assessed at 1 month and although this is therapeutically the most relevant window, late recovery up to 3 months may have reclassified some outcomes. The coarse three-level prediction scale, chosen to mirror clinical communication, limits resolution, and prompts were administered in Turkish to models trained predominantly on English corpora, so language effects cannot be excluded [14]. Finally, future studies (ideally prospective and multicenter) should include probabilistic outputs with formal calibration analysis, repeated fresh-session runs and alternative prompt formulations to quantify output variability, larger multi-institutional expert panels, and head-to-head comparison with supervised models on the same cohort; if the high positive predictive value of LLM “poor-prognosis” flags is confirmed, a pragmatic trial could test whether AI-supported early treatment intensification improves hearing outcomes in patients least likely to recover.

## 5. Conclusions

Freely available generative AI models, evaluated in a zero-shot, role-prompted manner on routine presentation data, predicted 1-month non-recovery from SSNHL with 70.0% accuracy—not significantly different from that of individual experienced otolaryngologists, although the study was not powered for equivalence testing, though below that of the best-performing expert (86.7%), and with a systematic tendency toward confident, correct rule-in but unreliable rule-out: in this cohort, every patient the models rated as having low recovery potential failed to recover, whereas high inter-model agreement (κw 0.658–0.667) did not translate into superior accuracy, showing that consensus among AI systems must not be mistaken for predictive validity. Together with the confirmation of baseline severity and treatment delay—and the hypothesis-generating association of BMI—as determinants of recovery, these findings define a specific, testable role for LLMs in SSNHL prognosis: the reliability of their “low recovery potential” ratings warrants prospective evaluation as a conservative signal for identifying patients who may benefit from closer follow-up and earlier counseling. The present study does not establish clinical effectiveness, and LLM predictions should not inform treatment or management decisions pending such validation; final judgment remains with the clinician. In settings where subspecialty neurotology expertise is not readily available, such models may eventually offer accessible decision support, provided their reliability is confirmed in larger, prospective, version-pinned evaluations with repeated runs and multiple prompt formulations.

## Figures and Tables

**Figure 1:**
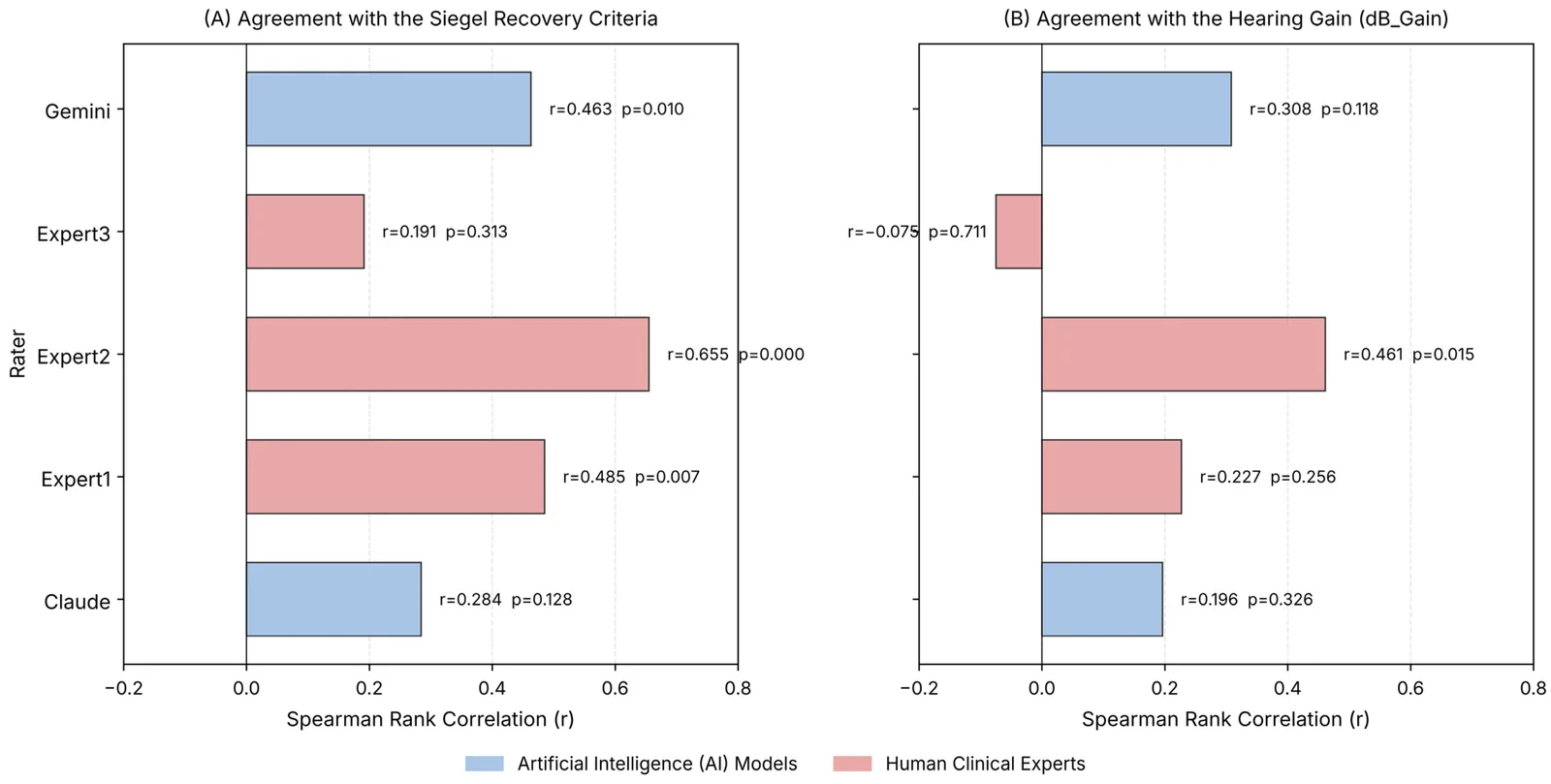
Comparison of prognostic prediction alignments between human clinical experts and artificial intelligence (AI) models. Panel (**A**) illustrates the Spearman rank correlation (r) of predictors with the objective Siegel recovery criteria. Panel (**B**) shows the correlation with the absolute hearing threshold gain (dB). Expert 2 demonstrated the highest clinical consistency across both outcomes, while Gemini outperformed other AI models, achieving prognostic alignment comparable to Expert 1.

**Figure 2:**
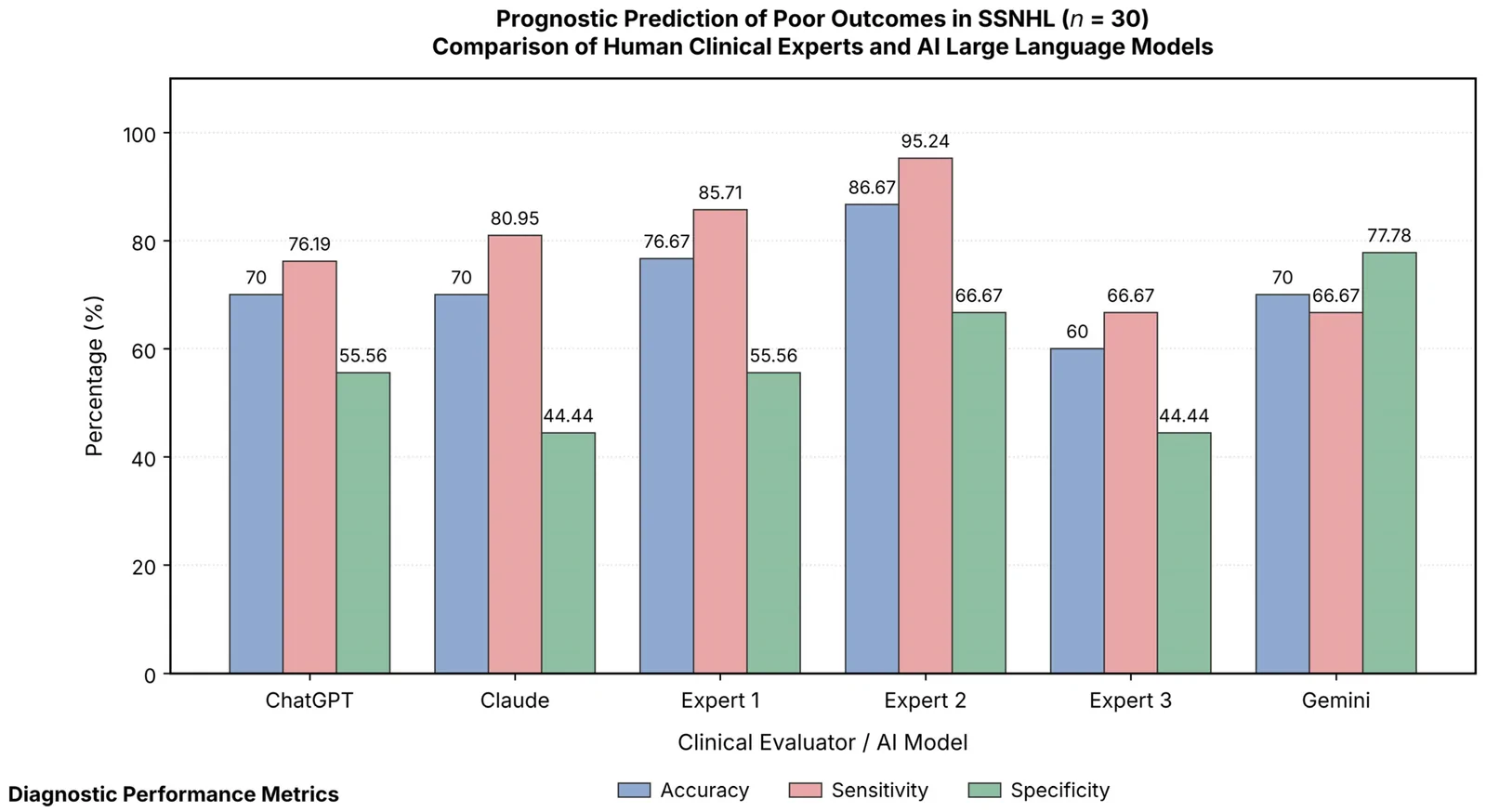
Grouped bar chart comparing the sensitivity, specificity, and overall accuracy of human clinical experts and AI large language models (*n* = 30) in predicting poor prognosis.

**Figure 3:**
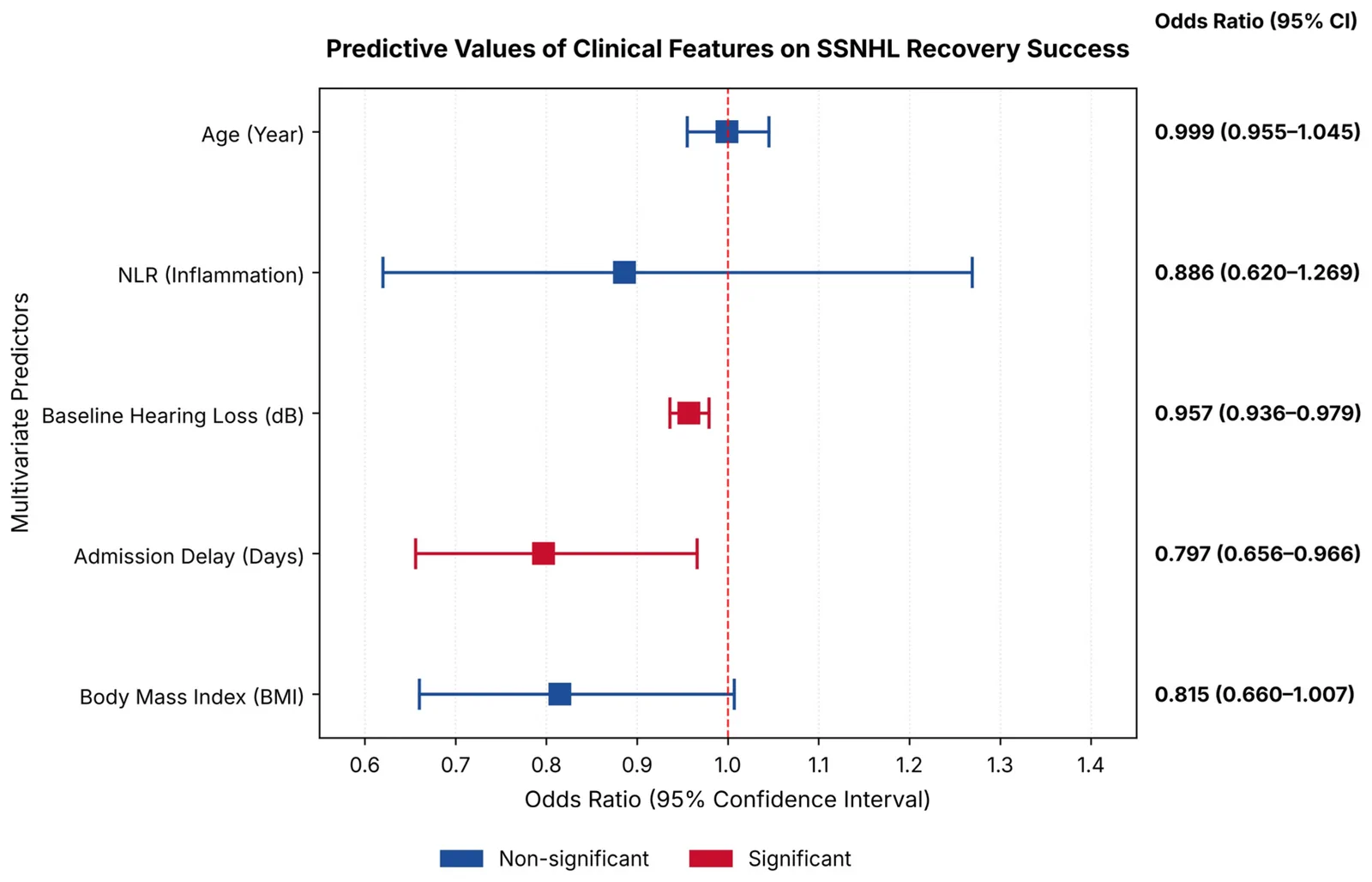
Forest plot of Odds Ratios from Multivariate Logistic Regression Analysis. The red dashed vertical line indicates the null reference (OR = 1.0).

**Figure 4:**
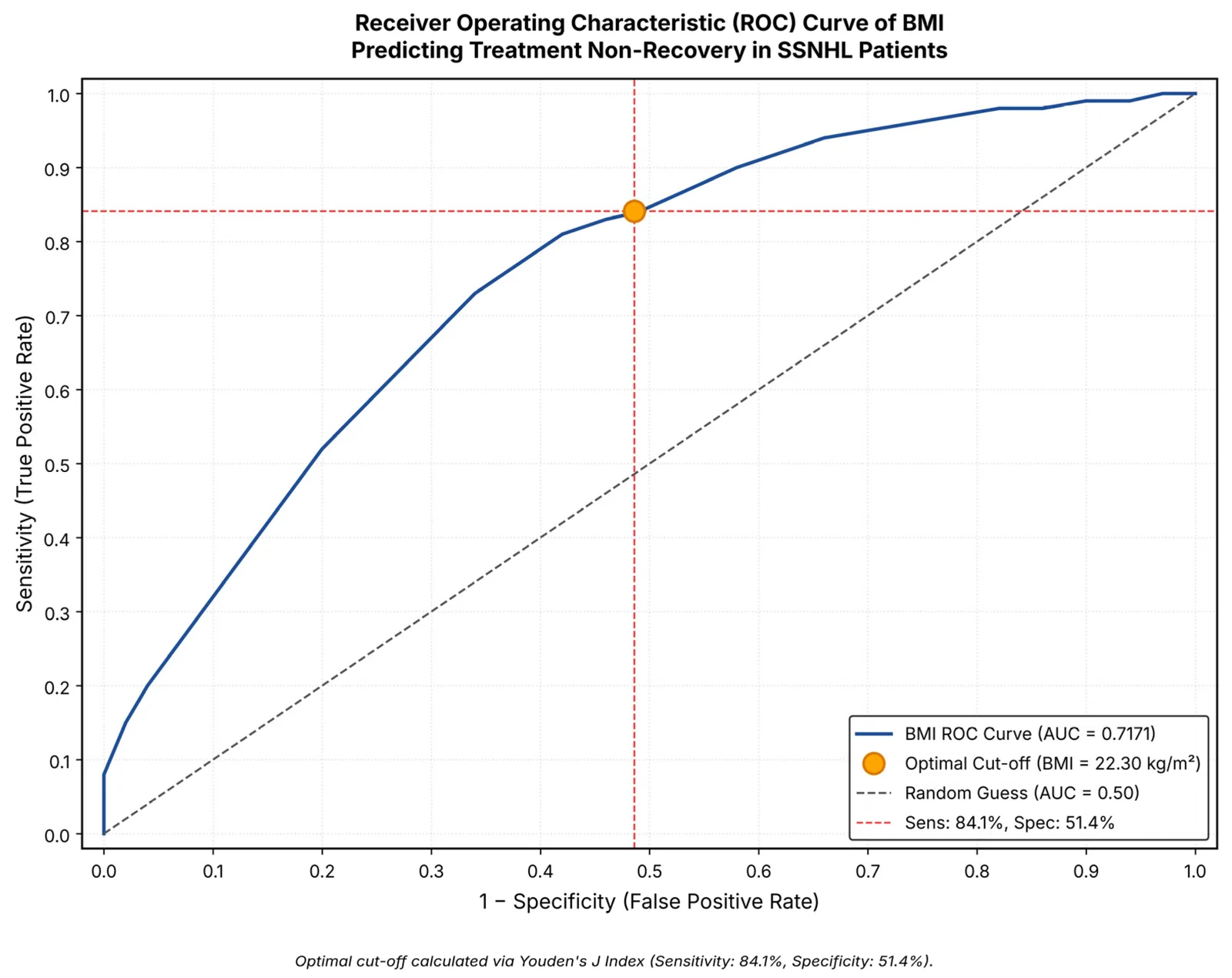
Receiver Operating Characteristic (ROC) curve of BMI predicting treatment non-recovery. The optimal cut-off (22.3 kg/m^2^) is highlighted by the orange dot (AUC = 0.7171, *p* = 0.0003).

**Figure 5:**
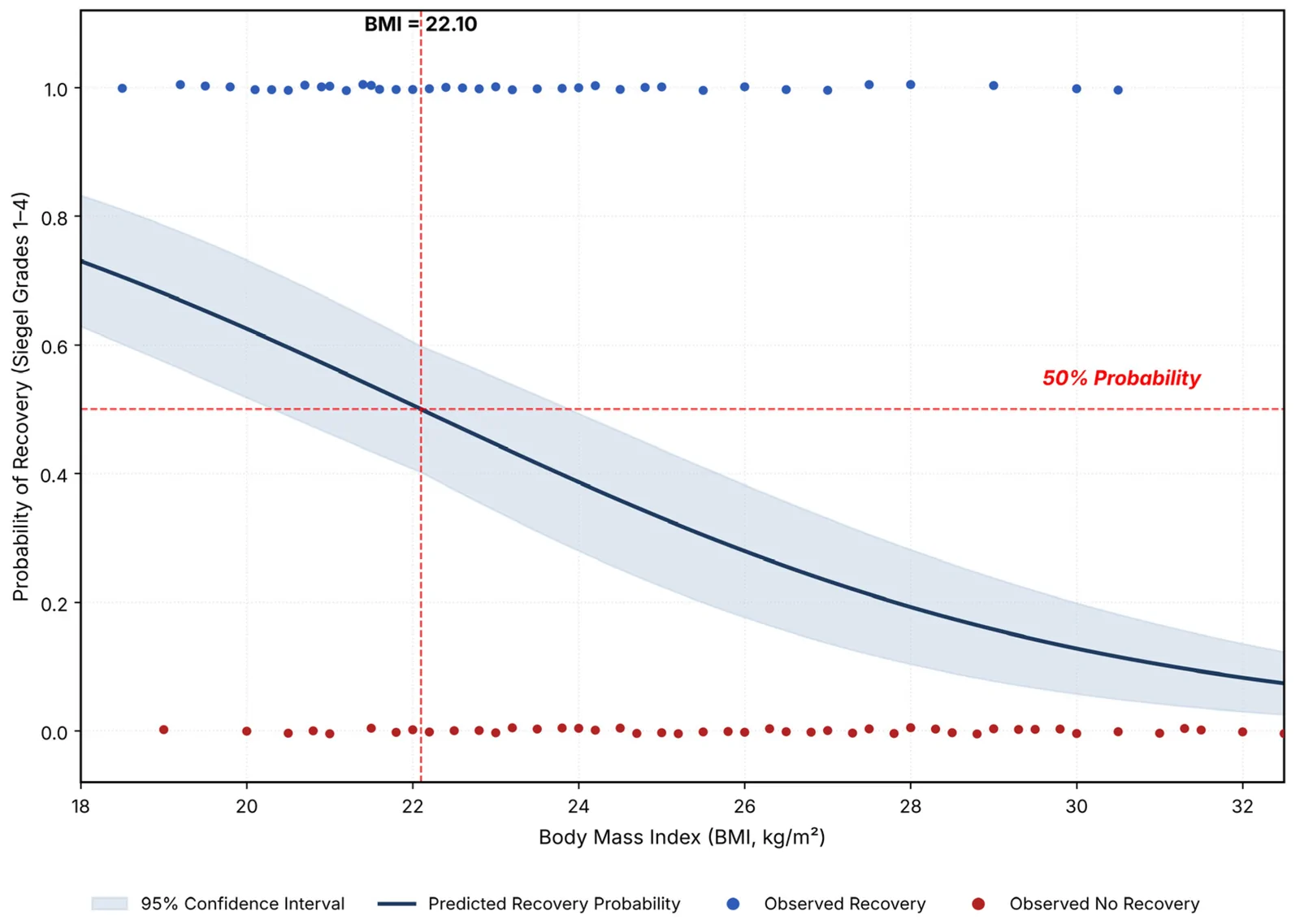
Logistic regression curve of the predicted probability of hearing recovery (complete or partial by Siegel criteria) as a function of body mass index (BMI). The shaded region indicates the 95% confidence interval; circles at y = 1 represent recovered and at y = 0 non-recovered patients. Dashed lines mark BMI = 22.1 kg/m^2^, where the predicted probability of recovery falls below 50%.

**Table 1:** Baseline characteristics of the observer sub-cohort (*n* = 30) and the full cohort (*N* = 100).

	Observer Cohort		Full Cohort	
Parameter	Value/Mean ± SD	Percentage/Range	Value/Mean ± SD	Percentage/Range
Age (Years)	48.2 ± 13.2	24.0–70.0	49.6 ± 13.8	10–74
Sex (Female/Male)	21/9	70.0%/30.0%	49/51	49.0%/51.0%
Body Mass Index (BMI, kg/m^2^)	24.1 ± 3.4	19.7–30.8	24.50 ± 3.30	18.3–32.4
Admission Delay (Days)	3.93 ± 3.33	1.0–16.0	4.8 ± 4.6	1–22
Baseline Hearing Severity (dB)	61.7 ± 24.3	28–120	62.2 ± 28.2	7–120
Hypertension Comorbidity (HT, Yes/No)	6/24	20.0%/80.0%	21/100	21.0%
Diabetes Mellitus Comorbidity (DM, Yes/No)	5/25	16.7%/83.3%	19/100	19.0%
Hyperlipidemia Comorbidity	9/21	30.0%/70.0%	44/100	44.0%
Overall Recovery at 1 Month (Siegel 1–3, complete or partial)	9/30	30.0%	37/100	37.0%
Neutrophil-to-Lymphocyte Ratio (NLR) ^†^	3.30 ± 4.72	0.99–26.6	2.71 ± 2.96	0.69–26.6
Low-Density Lipoprotein (LDL, mg/dL)	76.5 ± 39.96	36.0–173.0	82.2 ± 44.37	25.0–209.0

^†^ Available in 29 of 30 patients in the observer cohort and 99 of 100 in the full cohort.

**Table 2:** Matrix of Inter-Observer Linear Weighted Kappa (KW) agreement coefficients among the three human clinicians and three generative AI models across 30 sudden sensorineural hearing loss patients.

Evaluator	Expert 1	Expert 2	Expert 3	ChatGPT	Gemini	Claude
Expert 1	**1.000**	0.505	0.541	**0.720**	0.641	0.543
Expert 2	0.505	**1.000**	0.293	0.378	0.417	0.263
Expert 3	0.541	0.293	**1.000**	0.490	0.567	0.559
ChatGPT	**0.720**	0.378	0.490	**1.000**	0.667	0.658
Gemini	0.641	0.417	0.567	0.667	**1.000**	0.665
Claude	0.543	0.263	0.559	0.658	0.665	**1.000**

Matrix of Inter-Observer Linear Weighted Kappa (KW) agreement coefficients among the three human clinicians and three generative AI models across 30 sudden sensorineural hearing loss patients. The blue-shaded, bold value represent self-agreement (1.000) and diagonal references. The green-shaded, bold value (KW = 0.720) highlights the highest agreement between a human clinician and an AI model.

**Table 3:** Spearman Rank Correlation coefficients (r) and corresponding *p*-values between prognostic predictions (1–3 scale) and actual 1-month clinical outcomes (categorical Siegel Criteria and continuous hearing gain in decibels). Bold values marked with an asterisk (*) indicate statistical significance (*p* < 0.05).

Observer	Siegel Recovery Correlation (r)	*p*-Value (Siegel)	dB Gain Correlation (r)	*p*-Value (dB Gain)
**Expert 2**	0.655	*p* < 0.001 *	0.461	*p* = 0.015 *
**Expert 1**	0.485	*p* = 0.007 *	0.227	*p* = 0.256
**Gemini (AI)**	0.463	*p* = 0.010 *	0.308	*p* = 0.118
**ChatGPT (AI)**	0.373	*p* = 0.042 *	0.255	*p* = 0.200
**Claude (AI)**	0.284	*p* = 0.128	0.196	*p* = 0.326
**Expert 3**	0.191	*p* = 0.313	−0.075	*p* = 0.711

**Table 4:** Cross-tabulation of 3-level prognostic ratings by clinicians and AI models versus observed 1-month outcomes (*n* = 30). *p* values from the Fisher exact test.

Clinical Evaluator and Prognostic Category	Observed No Recovery (*n* = 21)	Observed Recovery (*n* = 9)	Total (*n* = 30)	*p*-Value
**Expert 1, *n* (%)**				**0.0152**
-Low Recovery Potential	10 (47.6%)	0 (0.0%)	10 (33.3%)	
-Moderate Recovery Potential	8 (38.1%)	4 (44.4%)	12 (40.0%)	
-High Recovery Potential	3 (14.3%)	5 (55.6%)	8 (26.7%)	
**Expert 2, *n* (%)**				**0.0006**
-Low Recovery Potential	9 (42.9%)	0 (0.0%)	9 (30.0%)	
-Moderate Recovery Potential	11 (52.4%)	3 (33.3%)	14 (46.7%)	
-High Recovery Potential	1 (4.8%)	6 (66.7%)	7 (23.3%)	
**Expert 3, *n* (%)**				0.3300
-Low Recovery Potential	8 (38.1%)	1 (11.1%)	9 (30.0%)	
-Moderate Recovery Potential	6 (28.6%)	4 (44.4%)	10 (33.3%)	
-High Recovery Potential	7 (33.3%)	4 (44.4%)	11 (36.7%)	
**ChatGPT, *n* (%)**				0.0859
-Low Recovery Potential	7 (33.3%)	0 (0.0%)	7 (23.3%)	
-Moderate Recovery Potential	9 (42.9%)	4 (44.4%)	13 (43.3%)	
-High Recovery Potential	5 (23.8%)	5 (55.6%)	10 (33.3%)	
**Gemini, *n* (%)**				**0.0304**
-Low Recovery Potential	10 (47.6%)	0 (0.0%)	10 (33.3%)	
-Moderate Recovery Potential	4 (19.0%)	2 (22.2%)	6 (20.0%)	
-High Recovery Potential	7 (33.3%)	7 (77.8%)	14 (46.7%)	
**Claude, *n* (%)**				0.1001
-Low Recovery Potential	7 (33.3%)	0 (0.0%)	7 (23.3%)	
-Moderate Recovery Potential	10 (47.6%)	5 (55.6%)	15 (50.0%)	
-High Recovery Potential	4 (19.0%)	4 (44.4%)	8 (26.7%)	

Bold values indicate statistical significance (*p* < 0.05). *p*-values were calculated using Fisher’s exact test.

**Table 5:** Diagnostic performance of clinicians and AI models in predicting non-recovery (*n* = 30).

Rater	Sensitivity, 95% CI	Specificity, 95% CI	Accuracy, 95% CI	PPV, 95% CI	NPV, 95% CI	McNemar vs. Exp2
Expert 2	95.2% (77.3–99.2)	66.7% (35.4–87.9)	86.7% (70.3–94.7)	87.0% (67.9–95.5)	85.7% (48.7–97.4)	Reference
Expert 1	85.7% (65.4–95.0)	55.6% (26.7–81.1)	76.7% (59.1–88.2)	81.8% (61.5–92.7)	62.5% (30.6–86.3)	*p* = 0.4497
Gemini	66.7% (45.4–82.8)	77.8% (45.3–93.7)	70.0% (52.1–83.3)	87.5% (64.0–96.5)	50.0% (26.8–73.2)	*p* = 0.2278
ChatGPT	76.2% (54.9–89.4)	55.6% (26.7–81.1)	70.0% (52.1–83.3)	80.0% (58.4–91.9)	50.0% (23.7–76.3)	*p* = 0.1824
Claude	81.0% (60.0–92.3)	44.4% (18.9–73.3)	70.0% (52.1–83.3)	77.3% (56.6–89.9)	50.0% (21.5–78.5)	*p* = 0.1824
Expert 3	66.7% (45.4–82.8)	44.4% (18.9–73.3)	60.0% (42.3–75.4)	73.7% (51.2–88.2)	36.4% (15.2–64.6)	*p* = 0.0269 *

* Statistically significant difference compared to Expert 2 (reference rater) by McNemar’s test (*p* < 0.05).

**Table 6:** Univariate comparison of demographic, clinical, and laboratory features (*N* = 100).

Predictor Variable	Favorable (*n* = 37)	Poor Outcome (*n* = 63)	*p*-Value
Patient Age (Years)	45.57 ± 13.26	52.02 ± 13.59	0.0117 *
Body Mass Index (BMI, kg/m^2^)	22.96 ± 3.05	25.41 ± 3.12	0.0003 *
Admission Delay (Days)	3.05 ± 2.79	5.78 ± 5.15	0.0016 *
Baseline Hearing Threshold (dB)	46.62 ± 23.17	71.33 ± 26.91	<0.0001 *
NLR (Inflammation)	2.22 ± 1.25	2.46 ± 1.64	0.8537

* Statistically significant difference between the favorable and poor outcome groups (*p* < 0.05).

**Table 7:** Multivariate logistic regression model predicting favorable hearing recovery (*N* = 99).

Predictor Variable	Adjusted OR	Lower 95% CI	Upper 95% CI	*p*-Value
Baseline Hearing (dB)	0.957	0.936	0.979	0.0001 *
Admission Delay (Days)	0.797	0.656	0.966	0.0211 *
Body Mass Index (BMI)	0.815	0.660	1.007	0.0586
NLR (Inflammation)	0.886	0.620	1.269	0.5100
Patient Age (Years)	0.999	0.955	1.045	0.9553

* Indicates statistical significance (*p* < 0.05).

## Data Availability

The datasets analyzed during the current study are available from the corresponding author on reasonable request.

## Supplementary Materials

The following supporting information can be downloaded at https://www.mdpi.com/article/10.3390/diagnostics16213646/s1, Supplementary Method S1: Standardized prompt used for the large language model (LLM) evaluations (Turkish original with English translation). Supplementary Figure S1: Comprehensive statistical model diagnostics for the multivariable logistic regression predicting favorable hearing recovery—Panel A (calibration plot with Hosmer–Lemeshow test), Panel B (ROC curve), Panel C (Cook’s distance), and Panel D (leverage versus Pearson residuals).
