# Supplementary material for: Large Language Models Versus Clinicians in Predicting Recovery from Idiopathic Sudden Sensorineural Hearing Loss
Source: Diagnostics (Basel). 2026 Nov 4;16(21):3646. doi: 10.3390/diagnostics16213646 (PMC13763414; doi:10.3390/diagnostics16213646)
Supplement: Supplementary file 1 [file diagnostics-16-03646-s001.zip › diagnostics-4591848-supplementary.pdf]

Supplementary Method S1. Standardized prompt used for the large language model evaluations.

Original prompt (Turkish):

"Kendini güncel literatürü takip eden, minimum 10 yıl tecrübeli bir KBB hekimi olarak düşün. Sana bazı hastalarının ani işitme kaybı sebebiyle başvurduktan sonra yapılan öykü sorgulamasından öğrenilen bilgiler ve bir takım laboratuvar sonuçlarını vererek bu hastaların ani işitme kayıplarının iyileşme ihtimalini predict etmeni istiyorum."

English translation:

"Act as an otolaryngologist with at least 10 years of experience who follows the current literature. I will provide the clinical and laboratory information of patients presenting with sudden hearing loss, obtained from history-taking after presentation, and ask you to predict the likelihood of recovery of their hearing loss."

For each of the 30 cases, the full structured variable set (as described in Section 2.2 of the main text) and the digital image of the baseline pure-tone audiogram were then supplied, and each model was asked to grade the likelihood of hearing recovery at 1 month as low (1), moderate (2), or high (3).

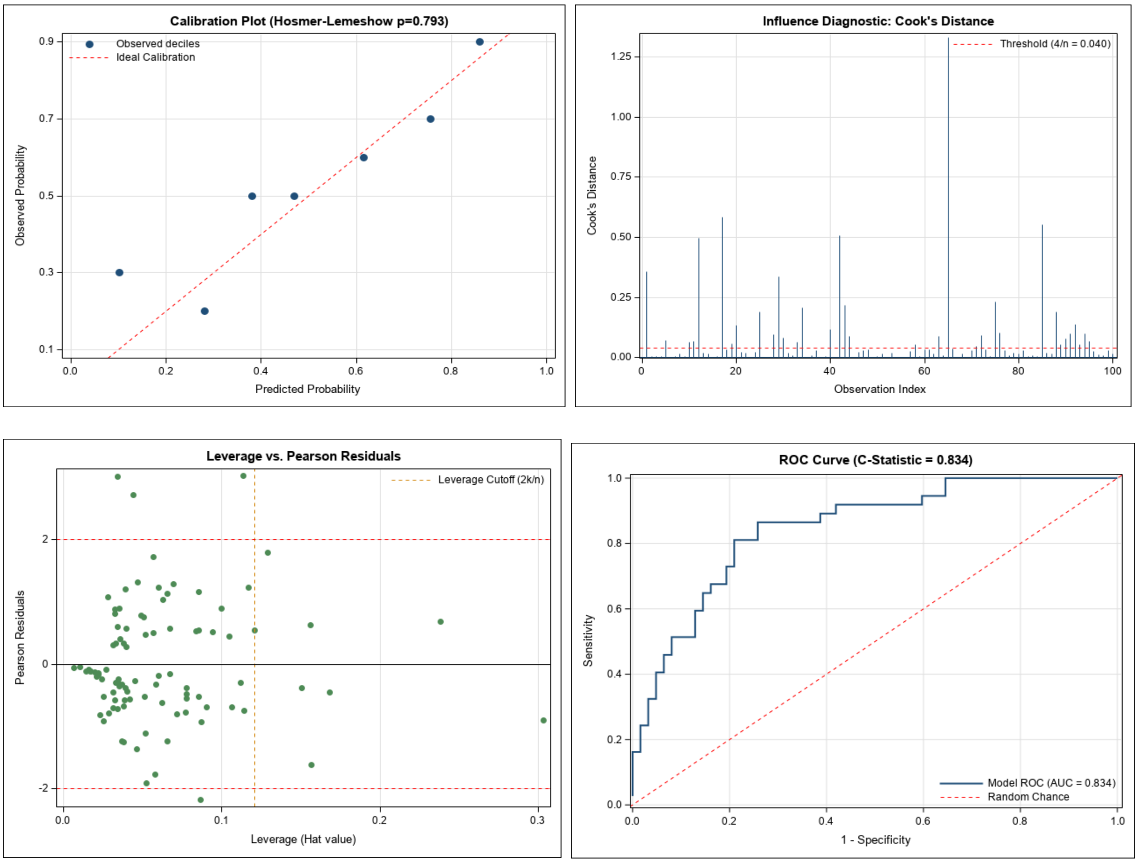

Supplementary Figure S1 Legend: Comprehensive statistical model diagnostics for the multivariable logistic regression predicting favorable hearing recovery (Siegel Grades 1 and 2) in the primary cohort (N = 99). Top-Left (Panel A): Model Calibration Plot displaying observed recovery outcomes versus predicted probabilities (P-hat), with a smoothed loess calibration curve overlapping the ideal diagonal (Hosmer-Lemeshow $\chi^2 = 3.1286$, df = 6, p = 0.7925), confirming excellent model calibration. Top-Right (Panel B): Receiver Operating Characteristic (ROC) curve showing high discriminative capacity with a C-statistic (AUC) of 0.8338 (95% CI: 0.748–0.920). Bottom-Left

(Panel C): Cook's Distance influence plot confirming no single influential observation exceeds the critical threshold (D_max = 0.1575 < 1.0). Bottom-Right (Panel D): Leverage versus Pearson Residuals plot demonstrating a balanced leverage distribution across all observations (h_max = 0.3144).
